# Saponin Formosanin C-Induced Ferritinophagy and Ferroptosis in Human Hepatocellular Carcinoma Cells

**DOI:** 10.3390/antiox9080682

**Published:** 2020-07-29

**Authors:** Pin-Lun Lin, Han-Hsuan Tang, Shan-Ying Wu, Ning-Sing Shaw, Chun-Li Su

**Affiliations:** 1 Department of Human Development and Family Studies, National Taiwan Normal University, Taipei 106, Taiwan; sp940921@gate.sinica.edu.tw; 2 Graduate Program of Nutrition Science, School of Life Science, National Taiwan Normal University, Taipei 106, Taiwan; 60751001s@ntnu.edu.tw (H.-H.T.); shanyingwu@tmu.edu.tw (S.-Y.W.); 3 Department of Microbiology and Immunology, School of Medicine, College of Medicine, Taipei Medical University, Taipei 110, Taiwan; 4 Department of Biochemical Science and Technology, College of Life Science, National Taiwan University, Taipei 106, Taiwan

**Keywords:** formosanin C, hepatocellular carcinoma, ferroptosis, autophagy, ferritinophagy

## Abstract

Ferroptosis, a recently discovered form of iron-dependent cell death, requires an increased level of lipid-reactive oxygen species (ROS). Ferritinophagy, a ferritin degradation pathway, depends on a selective autophagic cargo receptor (NCOA4). By screening various types of natural compounds, formosanin C (FC) was identified as a novel ferroptosis inducer, characterized by attenuations of FC-induced viability inhibition and lipid ROS formation in the presence of ferroptosis inhibitor. FC also induced autophagic flux, evidenced by preventing autophagic marker LC3-II degradation and increasing yellow LC3 puncta in tandem fluorescent-tagged LC3 (mRFP-GFP) reporter plasmid (ptfLC3) transfected cells when combined with autophagic flux inhibitor. It is noteworthy that FC-induced ferroptosis and autophagic flux were stronger in HepG2 cells expressing higher NCOA4 and lower ferritin heavy chain 1 (FTH1) levels, agreeing with the results of gene expression analysis using CTRP and PRISM, indicating that FTH1 expression level exhibited a significant negative correlation with the sensitivity of the cells to a ferroptosis inducer. Confocal and electron microscopy confirmed the pronounced involvement of ferritinophagy in FC-induced ferroptosis in the cells with elevated NCOA4. Since ferroptosis is a non-apoptotic form of cell death, our data suggest FC has chemotherapeutic potential against apoptosis-resistant HCC with a higher NCOA4 expression via ferritinophagy.

## 1. Introduction

Iron is an element that is critical for cell proliferation and growth. Besides these effects, iron creates reactive oxygen species (ROS) by participating in the Fenton reaction. Cellular iron metabolism comprises three major processes: iron uptake, storage, and export, which depend on three major proteins: transferrin receptor (TFRC), ferritin, and ferroportin (FPN), respectively. ROS-damaged DNA is associated with carcinogenesis. Several studies revealed that iron is highly demanded by cancer cells, as it facilitates the transformation and metastasis of tumor cells [1].

Ferroptosis is characterized by the iron-dependent accumulation of lipid ROS. Inhibition of cystine-glutamate antiporter (system X_c−_) causes the depletion of glutathione, especially glutathione peroxidase 4, a phospholipid hydroperoxidase that protects cells against membrane lipid peroxidation [2]. Thus, intracellular iron chelators (e.g., deferoxamine and desferrioxamine mesylate) and various antioxidants (e.g., vitamin E, liproxstatin-1, and ferrostatin-1 [Ferro-1]) prevent ferroptosis.

Autophagy is a eukaryotic cellular catabolic pathway that degrades cellular organelles and macromolecules via the lysosomes to recycle and protect cells at a basal state during stress [3]. Several investigations have provided robust data demonstrating that ferroptosis is an autophagy-related cell death process [4]. This process has been termed ferritinophagy, a form of selective autophagy, which contributes to ferroptosis by mediating the degradation of ferritin. Quantitative proteomics has revealed that nuclear receptor co-activator 4 (NCOA4) acts as the cargo receptor responsible for ferritin degradation by ferritinophagy [5].

Hepatocellular carcinoma (HCC), the second leading cause of cancer-related mortality worldwide, is characterized by considerable phenotypic and molecular heterogeneity. The co-existence of liver diseases in the majority of patients complicates the treatment and design of clinical trials. Current target therapy sorafenib only extends patients’ lives by about 3 months, so there is an urgent need for new interventions. It is noteworthy that the overexpression of ferritin has been observed in hepatocellular cancer, and FPN is significantly diminished in hepatocellular cancer cells as compared to their normal counterparts [6]. Therefore, it is reasonable to postulate that targeting high iron levels in cancer cells by inducing iron-dependent cell death may have therapeutic potential.

## 2. Materials and Methods

### 2.1. Reagents and Antibodies

Most of the chemicals were obtained from Sigma (St. Louis, MO, USA). Structurally defined, novel, and pure (>98.9% purity) garcinielliptone FC [7] and justicidin A [8] were gifts from Dr. Chun-Nan Lin (School of Pharmacy, Kaohsiung Medical University, Kaohsiung, Taiwan) and formosanin C (FC) was kindly donated by Dr. Shen-Jeu Won (College of Medicine, National Cheng Kung University, Tainan, Taiwan) [9]. Sorafenib (Nexavar^®^) was purchased from Bayer AG (Leverkusen, Germany). C11-BODIPY was obtained from Thermo Fisher Scientific Inc. (Waltham, MA, USA). Antibodies for Western blotting were purchased from the following vendors: rabbit monoclonal anti-CD71 (TFRC) and anti-ferritin heavy chain 1 (FTH1) antibodies, Cell Signaling Technology, Danvers, MA, USA; rabbit polyclonal anti-FPN/SLC40A1 antibody, Novus Biologicals, LLC, Littleton, CO, USA; rabbit polyclonal anti-ARA70 (NCOA4) antibody, Bethyl Laboratories, Inc., Montgomery, TX, USA; rabbit polyclonal anti-LC3B antibody, Abcam, Cambridge Science Park, Cambridge, UK; rabbit polyclonal anti-GAPDH antibody, GeneTex, Irvine, CA, USA; mouse monoclonal anti-β-actin antibody, Sigma; goat anti-rabbit (H+L) and goat anti-mouse IgM+IgG+IgA (H+L) HRP conjugate antibodies, Millipore Corp., Billerica, MA, USA.

### 2.2. Cell Culture

Human HCC cell lines Hep3B and HepG2 from American Type Culture Collection (Rockville, MD, USA) were grown at 37 °C in Dulbecco’s modified Eagle medium (GIBCO BRL, Gaithersburg, MD, USA) supplemented with penicillin and streptomycin as well as 10% fetal bovine serum (GIBCO) in a humidified atmosphere containing 5% CO_2_.

### 2.3. Database Analysis

The associations between cytotoxicity and gene expression were analyzed using the Cancer Therapeutics Response Portal (CTRP, https://portals.broadinstitute.org/ctrp/) and Profiling Relative Inhibition Simultaneously in Mixtures (PRISM, https://depmap.org/portal/prism/), both of which were developed by the Broad Institute (Cambridge, MA, USA) of Harvard University and Massachusetts Institute of Technology. CTRP links genetic, lineage, and other cellular features of cancer cell lines to the sensitivity of small molecules for effectively identifying patient-matched cancer therapeutics. PRISM was developed for high-throughput multiplexed screening >750 genomically characterized human cancer cell lines which can represent more than 45 lineages.

### 2.4. Measurement of Cell Viability Inhibition

The modified colorimetric 3-[4-5-Dimethylthiazol-2-yl]-2,5-diphenyltetrazolium bromide (MTT) assay [10] was used to determine the viability of the cells. After treatment, the Dulbecco’s modified Eagle medium containing drugs was replaced to avoid color interference. MTT was added and the absorbance was determined at 590 nm in an ELISA Reader (Synergy HT, BioTek, Highland Park, Winooski, VT, USA). The sulforhodamine B (SRB) assay was also used to determine cell density based on the cellular protein content [11]. After treatment, the cells were fixed with 10% trichloroacetic acid, and the cells were then stained with 0.1% (*w*/*v*) SRB dissolved in 1% acetic acid (J. T. Baker, Center Valley, PA, USA) for 1 h. The unbound dye was eliminated by washing with 1% acetic acid. The protein-bound dye was dissolved with 20 mM (pH = 10) Tris (Bionovas Biotechnology Co., Ltd., Toronto, Ontario, Canada) for determination at 540 nm using an ELISA reader.

### 2.5. Measurement of Autophagy Level by Flow Cytometry

Cells containing acidic vesicular organelles (AVOs), which represent the formation of autolysosomes at the later stage of autophagic flux [12], were determined by flow cytometry. The samples were collected and stained with acridine orange (1.5 µg/10 mL). After incubating in the dark at room temperature for 15 min, the suspended cells were transferred to a 5 mL FACS tube and then analyzed on a flow cytometer (LSRFortessa, Becton Dickinson, Lexington, KY, USA). Data were evaluated using ModFit LTTM and WinMDI 2.8 (Windows Multiple Document Interface Flow Cytometry Application).

### 2.6. Western Blot Analysis

Whole cell lysates were extracted in M-PER lysis buffer, and the protein levels were quantified with protein assay dye (Bio-Rad Laboratories, Hercules, CA, USA). The protein samples were then run on 8–10% SDS-PAGE gels and transferred onto PVDF membranes (Perkin Elmer, Santa Clara, CA, USA). After blocking with 5% milk in TBST according to the antibody manufacturer’s suggestions, HRP secondary antibody was added. Detection of protein signals were performed with chemiluminescent HRP substrate reagents (Millipore Corp.).

### 2.7. Measurement of Cellular Lipid ROS by Flow Cytometry

Lipid ROS generation was measured by flow cytometry. The collected samples were stained with 10 µM of C11-BODIPY in the dark at 37 °C for 30 min. The suspended cells were transferred to a 5 mL FACS tube and determined on a flow cytometer (LSRFortessa, Becton Dickinson, Lexington, KY, USA).

### 2.8. Transmission Electron Microscopy

After fixation and dehydration, the samples were incubated with propylene oxide prior to exposure to propylene oxide/Epikote [13,14]. The blocks were embedded with Epon-Araldite mixture (Electron Microscopy Sciences, Hatfield, PA, USA) and sectioned. The sliced samples were then examined under a transmission electron microscope (HITACHI-7000, Hitachi, Japan). For immunogold labeling, the sections of the cells on nickel grids were treated with 10% H_2_O_2_ for 10 min and then blocked with SuperBlock^TM^ blocking buffer (Thermo Fisher Scientific Inc.) for 30 min followed by mouse monoclonal anti-FTH1 (Santa Cruz Biotechnology, Inc., Dallas, TX, USA) and rabbit polyclonal anti-NCOA4 (Abcam) antibody treatment overnight. The grids were hybridized with goat polyclonal anti-mouse (20 nm) and goat polyclonal anti-rabbit (12 nm) secondary antibodies containing gold particles (Abcam) for 1 h. The sections were then stained with saturated uranyl acetate and lead citrate. The images were examined under a transmission electron microscope.

### 2.9. Confocal Microscopy

After treatment, cells fixed with paraformaldehyde were permeabilized with 0.1% Triton X-100 [15]. After washing with TBST and blocking with 5% BSA, the cells were stained with mouse monoclonal anti-FTH1 (Santa Cruz Biotechnology, Inc.) or anti-NCOA4 antibody in combination with rabbit polyclonal anti-LC3 (Abcam) antibody. After washing, the cells were stained with Alexa Fluor^®^ 568 goat anti-mouse IgG and Alexa Fluor^®^ 488 goat anti-rabbit IgG (Invitrogen, Carlsbad, CA, USA). Nuclei were visualized by incubating the cells with DAPI. The signal was examined using a confocal microscope (Zeiss LSM780, Zeiss, Oberkochen, Germany). For autophagic flux study, cells were transfected with tandem fluorescent-tagged LC3 (mRFP-GFP) reporter plasmid (ptfLC3) [16]. The signal was examined using fluorescence microscopy (Olympus BX61, Tokyo, Japan).

### 2.10. Statistical Analysis

All data were generated from at least three independent experiments. The data are presented as means ± standard errors of the means (SEMs). Student’s t-test and one-way ANOVA were used for statistical analysis with *p* < 0.05 considered to be statistically significant.

## 3. Results

### 3.1. FC Induced Stronger Ferroptosis in HepG2 Cells Compared to Hep3B Cells

**Figure 1:**
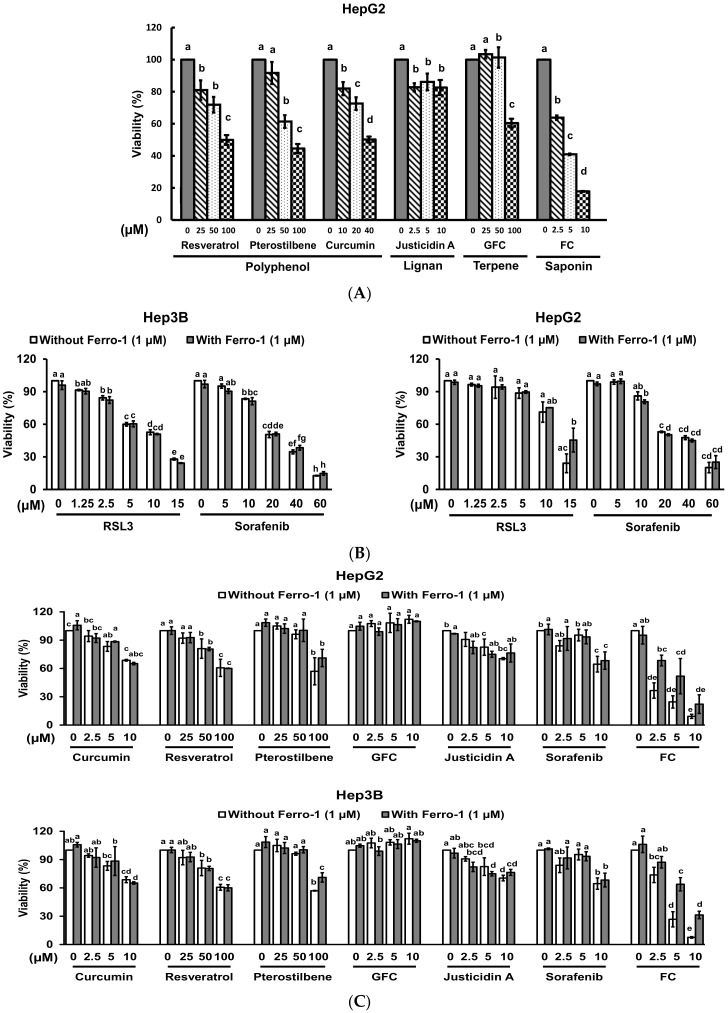

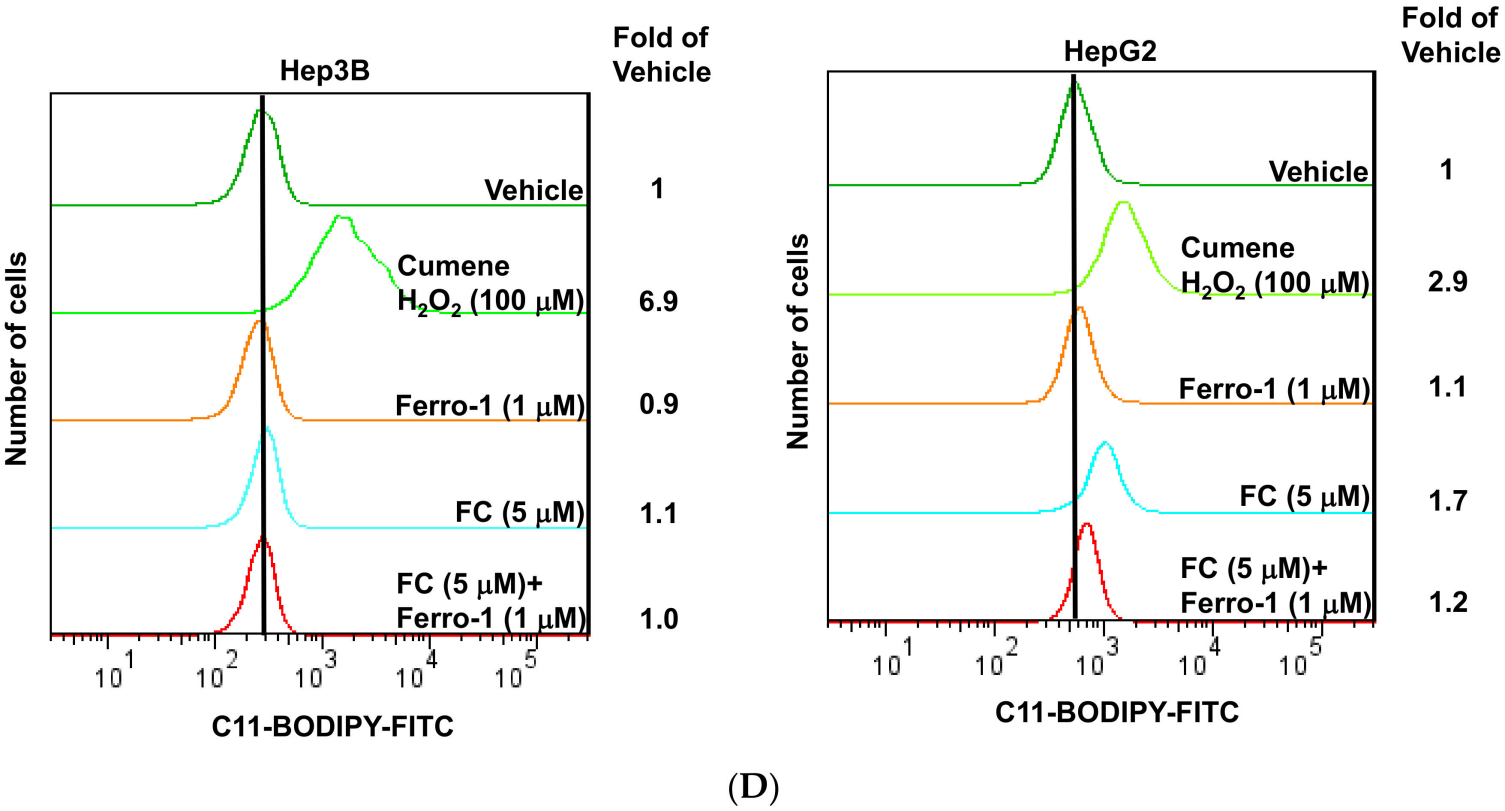
Formosanin C (FC)-induced ferroptosis was more effective in HepG2 cells. (**A**) Viability inhibition by various types of natural phytochemicals. HepG2 cells were treated with the indicated concentrations of sorafenib, resveratrol, pterostilbene, garcinielliptone FC (GFC), curcumin, justicidin A, or FC. After 48 h of incubation, the viability of the cells was evaluated by MTT assay. (**B**) Ferroptosis inducer RSL3- and sorafenib-triggered ferroptosis. (**C**) Phytochemical-induced ferroptosis was reversed by ferroptosis inhibitor. Hep3B and HepG2 cells were treated with various kinds of phytochemicals or anti-cancer drug sorafenib in the presence and absence of Ferro-1 for 24 h. Ferroptosis inducer RSL3 was also used. The viability of both cell lines was measured by SRB assay. The data are expressed as means ± SEMs. Means within a compound with different superscript letters are significantly different, *p* < 0.05. (**D**) FC-induced lipid ROS was partially reversed by ferroptosis inhibitor. After 24 h of treatment, the cells were stained with C11-BODIPY before flow cytometry. Cumene H_2_O_2_ was used as a positive control. The shift of the peak to the right indicates an increase in lipid ROS. The vertical line across the peak of vehicle is to illustrate the shifting of the peak. FC denotes formosanin C. GFC denotes garcinielliptone FC.

Although the health benefits of phytochemicals have been ascribed to their antioxidant and free radical quenching properties [17], certain phytochemicals also exhibit pro-oxidant activities and enhance the efficacy of certain cancer treatments [18]. To identify natural compounds that have the potential to induce ferroptosis, human HCC HepG2 cells were treated with different kinds of phytochemicals for evaluating the viability of the cells. As shown in Figure 1A, all tested phytochemicals suppressed the viability of the cells in a dosage-related manner. Among them, a diosgenin saponin FC displayed the strongest cytotoxicity. To determine if ferroptosis was involved in the FC-induced viability inhibition, both HCC Hep3B and HepG2 cells were co-treated with ferroptosis inhibitor Ferro-1 (a lipid ROS scavenger) [19] and each of the phytochemicals. Sorafenib, a U.S. Food and Drug Administration-approved targeted therapy for advanced HCC, and ferroptosis inducer RSL3 [20] were also used. As shown in Figure 1B, RSL3 and sorafenib separately exhibited cytotoxicity in both Hep3B and HepG2 cells in a dosage-related manner. The viability inhibition induced by RSL3 in HepG2 cells was partially rescued by Ferro-1, but the phenomenon was not observed in Hep3B cells, suggesting that HepG2 cells were more sensitive to ferroptosis compared to Hep3B cells. Sorafenib also suppressed the viability of both Hep3B and HepG2 cell lines, while no attenuation was observed in both cell lines. It is noteworthy that the cytotoxicity of FC on both cell lines was much greater than that of Sorafenib, and the FC-induced viability inhibition was significantly reversed by the presence of Ferro-1. Moreover, a lower dosage of FC (2.5 μM) was sufficient to induce significant ferroptosis in HepG2 cells compared to that in Hep3B cells (Figure 1C).

The ferroptotic cell death mechanism occurs via a lipid ROS-dependent process [21], thus FC-induced ferroptosis was confirmed by the formation of lipid ROS. In agreement with the cytotoxicity results (Figure 1C), FC-induced lipid ROS was more effectively reversed in HepG2 cells by the presence of Ferro-1 (Figure 1D). These data indicate that HepG2 cells were more sensitive to FC-induced ferroptosis compared to Hep3B cells.

### 3.2. FC-Induced a Higher Degree of Autophagic Flux in HepG2 Cells

**Figure 2:**
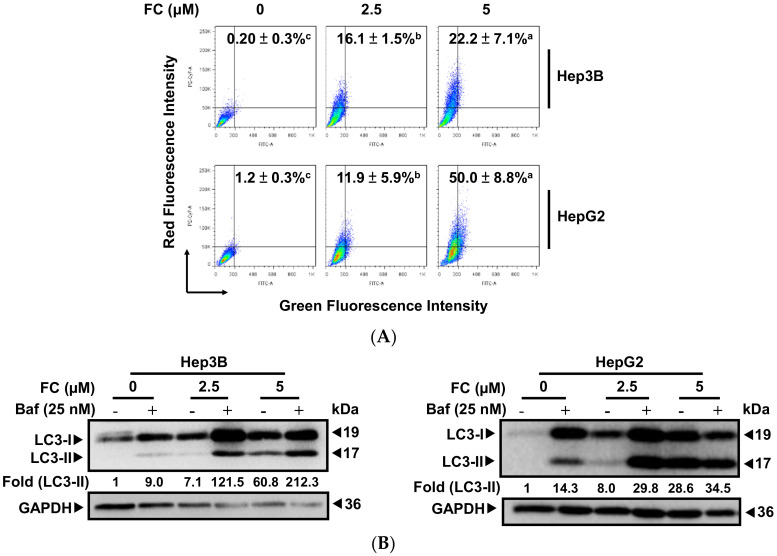

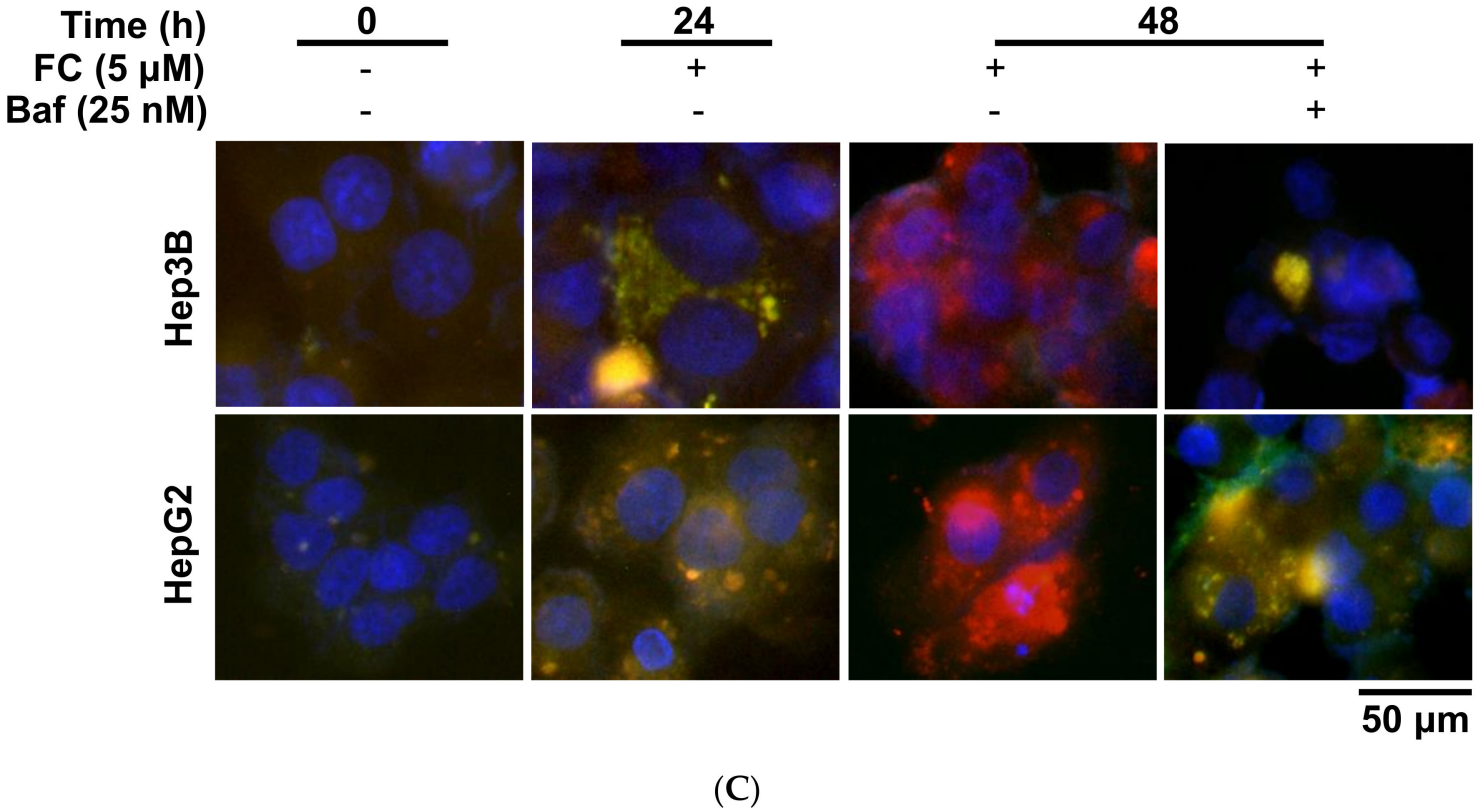
FC-induced autophagy was more effective in HepG2 cells. (**A**) FC induced a stronger degree of autophagy flux in HepG2 cells using flow cytometry. After 48 h of treatment, cells were stained with acridine orange, and 10,000 gated cells of each condition were used for flow cytometric analysis. The percentages in the figure indicate the proportion of cells with AVOs staining (upper two quadrants). The data are presented as means ± SEMs. Means within a cell line with different superscript letters are significantly different, *p* < 0.05. (**B**) FC induced a higher degree of autophagy in HepG2 cells using Western blotting. Cells were pretreated with autophagy flux inhibitor Baf for 2 h, and then treated with FC for 24 h. Whole-cell lysates were subjected to Western blot analysis using anti-LC3 antibody. GAPDH served as a loading control. The intensity of each protein expression band was quantified through densitometry normalization to that of GAPDH, with the control level arbitrarily set to 1. (**C**) FC induced autophagic flux. Cells transfected ptfLC3 plasmid were pretreated with Baf for 2 h and then treated with FC for 24 and 48 h. DNA morphologies were visualized by incubating the cells with DAPI. The signal was examined using fluorescence microscopy.

Autophagy is a lysosome-dependent degradation pathway. Autophagic flux describes the whole process of autophagy from the formation of autophagosomes to the breakdown of macromolecules in the autolysosomes. Impaired autophagic flux is involved in a variety of human pathophysiological processes, including cancer [22]. Recently, ferroptosis has been reported to be a form of autophagy-related cell death [4] via degradation of the iron storage protein ferritin (ferritinophagy) [23], which is mediated by the cargo receptor NCOA4 [5,24,25,26]. To determine if autophagy is involved in FC-induced ferroptosis, the formation of AVOs was examined using flow cytometry. As shown in Figure 2A, AVOs levels were significantly increased in both cell lines when the dosage of FC increased. It is noteworthy that FC triggered autophagy in HepG2 cells to a greater extent compared to that in Hep3B cells, indicating that FC-induced autophagy was more effective in HepG2 cells. The FC-induced autophagic flux was confirmed using Western blotting. As LC3-II stays with autophagosomes until the formation of autolysosomes, it has been used as a marker of autophagy [27]. As shown in Figure 2B, the expression of LC3-II rose in both cell lines as the FC dosage increased. Consistent with the results obtained using flow cytometry (Figure 2A), a higher expression of LC3-II was observed in FC-treated HepG2 cells. To further confirm if FC indeed induced autophagic flux, bafilomycin A1 (Baf) was added to inhibit the fusion between autophagosomes and lysosomes [28]. As shown in Figure 2B, the levels of LC3-II increased in both FC-treated cell lines in the presence of Baf compared to the cells treated with FC alone, and this elevation was more prominent in HepG2 cells compared to that in Hep3B cells. Moreover, the level of LC3-II was also increased in the cells treated with Baf alone (in the absence of FC), and a greater elevation was observed in HepG2 cells compared to that in Hep3B cells. These results suggest that HepG2 cells exhibited greater basal autophagy and were more sensitive to FC-induced autophagy compared to Hep3B cells.

To confirm FC-induced autophagy flux, a tandem fluorescent-tagged LC3 (mRFP-GFP) reporter plasmid (ptfLC3) was transfected into Hep3B and HepG2 cells [16]. The GFP-LC3 loses fluorescence intensity due to the acidic and degradative conditions in autolysosomes, while mRFP is resistant. Thus, the GFP and mRFP signals were detected before fusing with lysosomes (merged image exhibits yellow fluorescence), but only the mRFP signal could be detected after the fusion (merged image exhibits red fluorescence) [29]. As shown in Figure 2C, FC induced red puncta in the merged image, indicating the formation of autolysosomes. It is noteworthy that Baf changed the puncta color to yellow in the FC-treated cells, suggesting the accumulation of autophagosomes due to the prevention of FC-induced autophagy. Taken together, these observations demonstrated that FC indeed induced autophagy flux in Hep3B and HepG2 cells.

### 3.3. Cells Which Displayed a Higher Expression Level of NCOA4 were More Susceptible to Ferritinophagy

**Figure 3:**
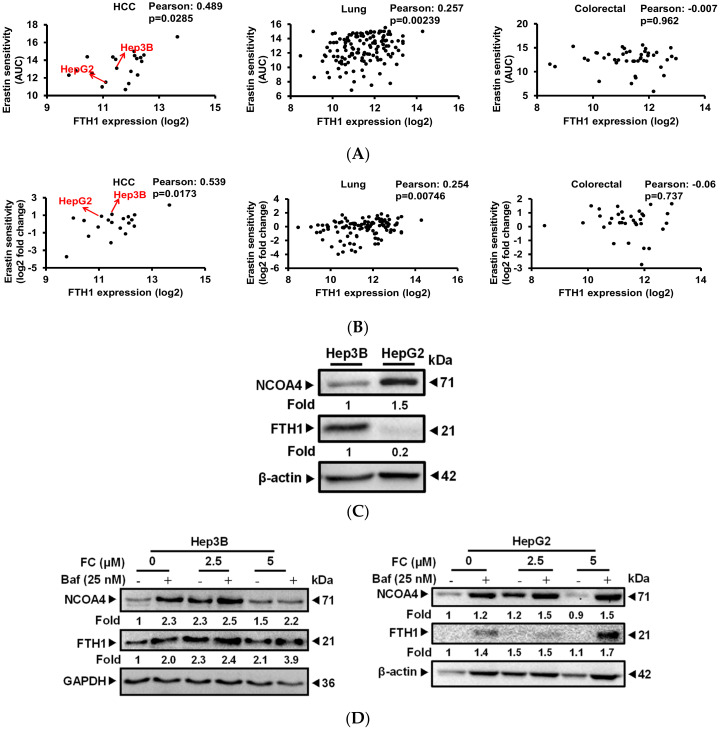

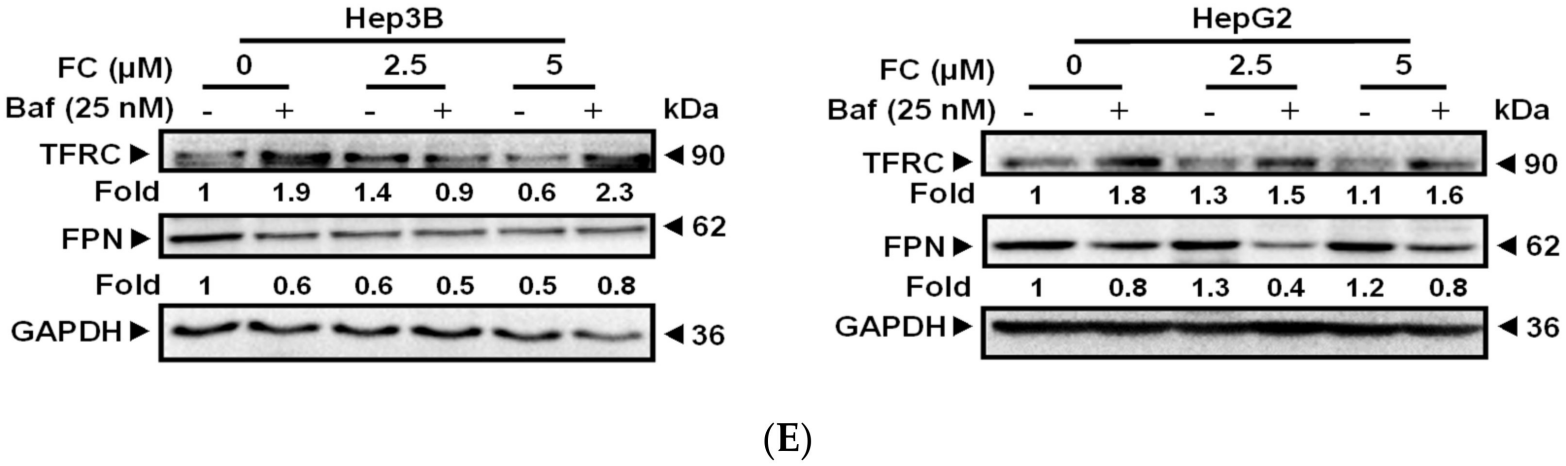
FC induced a stronger ferritinophagy in HepG2 cells. (**A**) Gene analysis results using CTRP indicate the FTH1 expression level in HCC exhibited a significant negative correlation with the sensitivity. (**B**) Gene analysis results using PRISM exhibited the same correlation observed using CTRP. AUC denotes the value of the area under the dose-response curve. Pearson correlation coefficient > 0.4 and < −0.4 considered to be statistically significant in positive and negative correlation with AUC, respectively. The lower the AUC value, the higher the sensitivity. (**C**) Opposite expression pattern of NCOA4 and FTH1 in Hep3B and HepG2 cell lines. (**D**) FC induced a stronger NCOA4 degradation in HepG2 cells. (**E**) Expression of iron metabolism proteins. Cells were pretreated with Baf for 2 h, and then treated with FC for 24 h. Whole-cell lysates were subjected to Western blot analysis using anti-NCOA4, anti-FTH1, anti-TFRC, and anti-FPN antibodies. GAPDH or β-actin served as a loading control. The intensity of each protein expression band was quantified through densitometry normalization to that of GAPDH or β-actin, with the control level arbitrarily set to 1.

To reveal the possible mechanisms which would explain the differences in the sensitivity of FC-induced ferroptosis between HepG2 and Hep3B cell lines, gene expression database analysis was carried out. Results from both CTRP (Figure 3A) and PRISM (Figure 3B) indicate that HepG2 has a lower mRNA expression level of FTH1 compared to Hep3B cells, and the FTH1 expression level in HCC (the most common type of primary liver cancer) exhibited a significantly (Pearson correlation coefficient > 0.4) negative correlation with the sensitivity (the lower the AUC value, the higher the sensitivity) of the cells to erastin, a ferroptosis inducer [30]. It is noteworthy that such a phenomenon was not exhibited in other cancer origins, including lung or colon cancers in CTRP (Figure 3A) or PRISM (Figure 3B). Figure 3C presents data confirming FTH1 expression and further shows that these two HCC cell lines (Hep3B and HepG2) also exhibit a distinct expression pattern of NCOA4. HepG2 cells not only express a lower level of FTH1 but also a higher level of NCOA4. NOCA4 was reported to act as a ferritinophagy-specific cargo receptor and was shown to play an important role in the degradation of the iron storage protein ferritin [5]. A lower expression level of FTH1 in HepG2 cells may be associated with higher basal autophagy in the cells (Figure 2). To determine if the expression pattern could discriminate the degree of FC-induced ferritinophagy in these two cell lines, the expression of NCOA4 and FTH1 was evaluated in the presence and absence of autophagy flux inhibitor Baf. As shown in Figure 3D, in the presence of Baf, the levels of both NCOA4 and FTH1 were elevated to a greater extent in FC (5 μM)-treated HepG2 cells compared to that in FC (5 μM)-treated Hep3B cells. Although Baf has been reported to interrupt transferrin flux [31], Baf caused similar and an even greater accumulation of FTH1 in FC (5 μM)-treated Hep3B and HepG2 cells, respectively, compared to the corresponding FC-untreated control (Figure 3D), suggesting that FC alone indeed induced degradation of FTH1 in both cell lines. Taken together, these data indicate that ferritinophagy proceeded more effectively in FC-treated HepG2 cells, which exhibit higher expression of ferritinophagy cargo receptor NCOA4.

Intracellular iron levels are actively regulated by TFRC and FPN, which transport iron into and out of the cells, respectively. In both lines of FC-treated cells, TFRC expression was increased, and co-administration of Baf further elevated the expression levels (Figure 3E). However, these phenomena were not observed for FPN expression (Figure 3E). These results suggest that FC may increase iron levels in cells by up-regulating TFRC expression, and Baf may inhibit both autophagy flux and transferrin flux causing iron deficiency and further increasing the expression of TFRC.

### 3.4. Confirmation of FC-Induced Ferritinophagy and Ferroptosis

**Figure 4:**
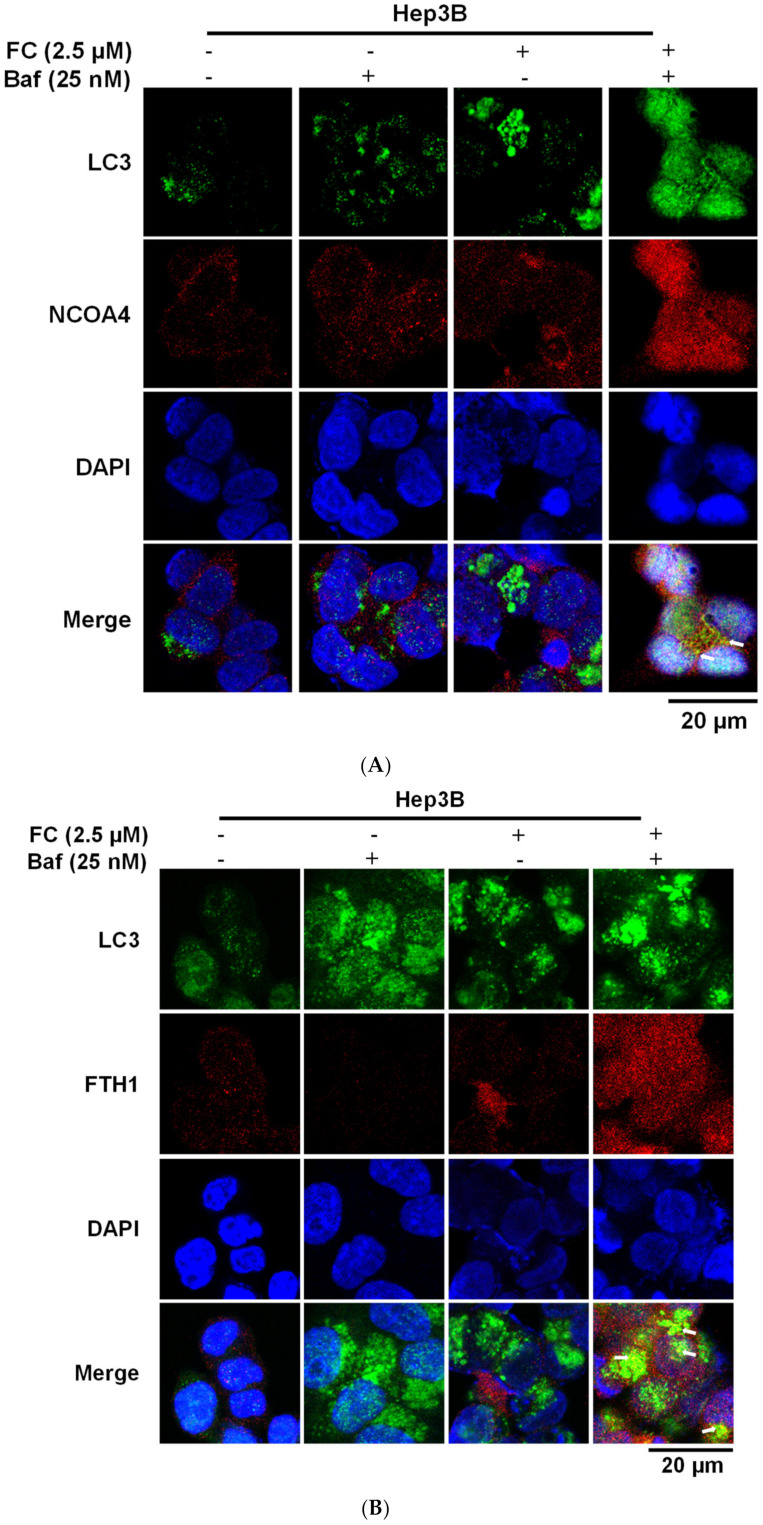

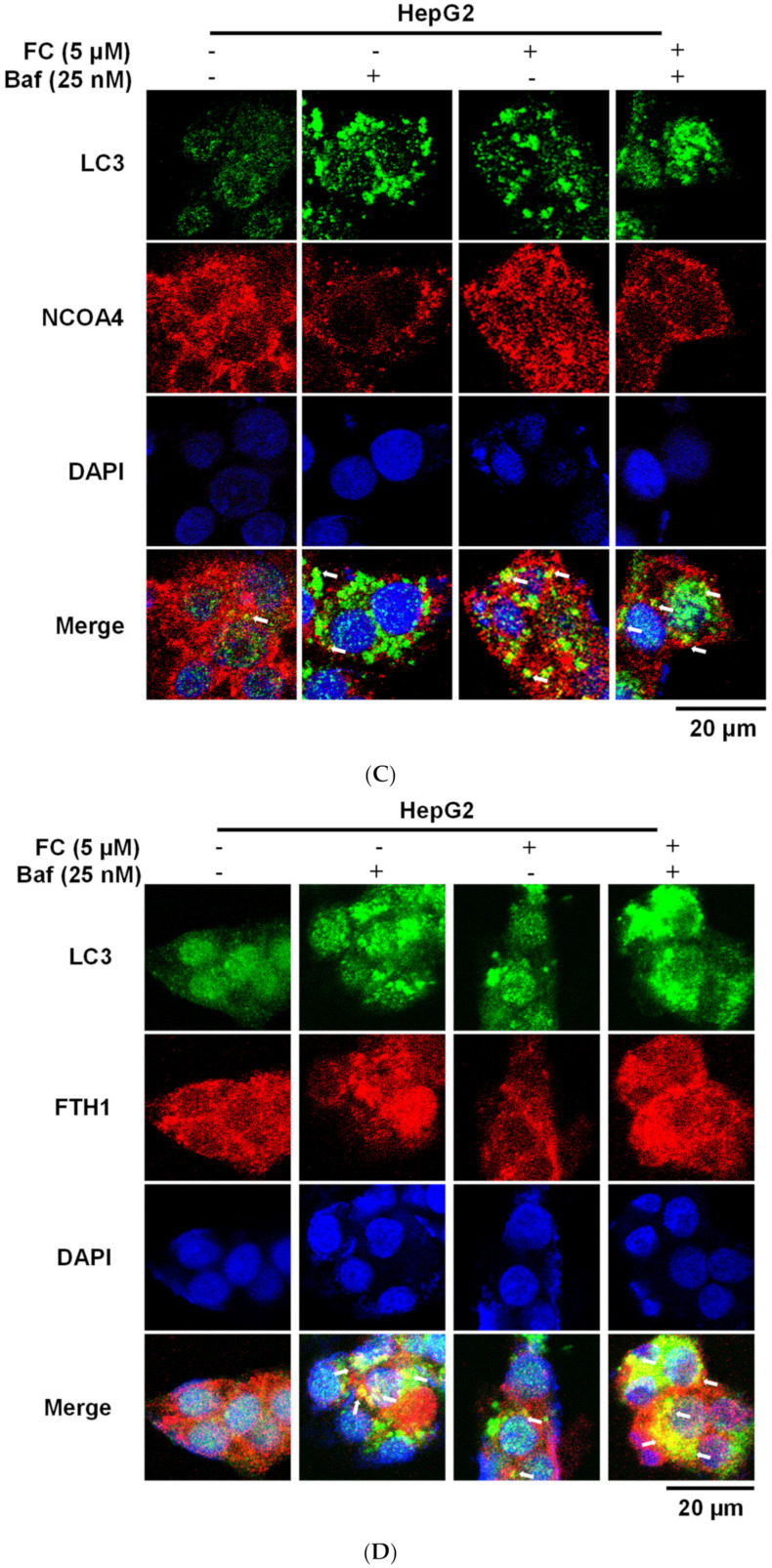

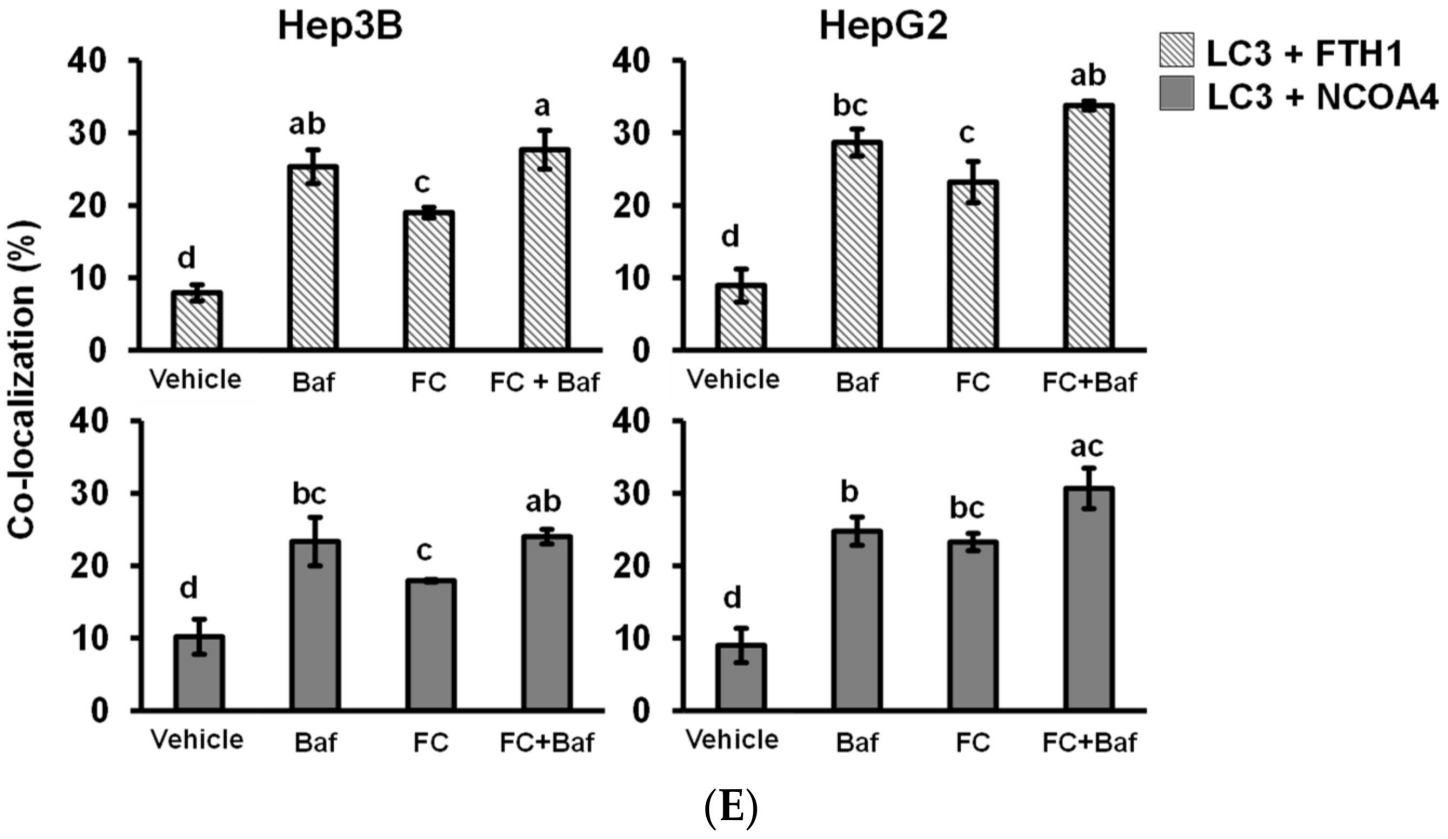
LC3 partly co-localized with NCOA4 and FTH1. (**A**) FC-induced co-localization of LC3 and NCOA4 in Hep3B cells. (**B**) FC-induced co-localization of LC3 and FTH1 in Hep3B cells. (**C**) FC-induced co-localization of LC3 and NCOA4 in HepG2 cells. (**D**) FC-induced co-localization of LC3 and FTH1 in HepG2 cells. (**E**) Quantitation of co-localization. The percentage of colocalization was quantified by counting the yellow dots in the cells from three fields of 30 cells in each experimental condition. The data are expressed as means ± SEMs. Means of FTH1 or NCOA4 of a cell line with different superscript letters are significantly different, *p* < 0.05. Cells were pretreated with autophagy flux inhibitor Baf for 2 h and then treated with FC for 24 h. Cells were then stained with anti-NCOA4 (red) or anti-FTH1 (red) antibody in the presence of DAPI (blue) and anti-LC3 (green) antibodies to visualize NCOA4, FTH1, cell nuclei, and autophagic puncta pattern of LC3, respectively. The images were taken by confocal microscopy. The arrow in the merge images indicates yellow puncta of LC3 (green) with NCOA4 (red) or FTH1 (red).

During autophagy progression, LC3-I is converted to a lipidated form of LC3-II, which leads to its translocation from the cytosol to the phagophores/autophagosomes [32], appearing as bright puncta under a fluorescent microscope [33]. To examine the FC-induced ferritinophagy, the co-localization of the LC3 puncta with NCOA4 or FTH1 was determined. Since the blockage of NCOA4 and FTH1 by Baf was more apparent in FC-treated Hep3B and HepG2 cell lines at the concentration of 2.5 and 5 μM (Figure 3D), respectively, these concentrations were used to study FC-induced ferritinophagy. As shown in Figure 4A–E, an increase in the intensity of LC3 puncta was observed in both cell lines treated with Baf or FC alone, suggesting that basal autophagy existed and FC was able to induce autophagy in both cell lines, respectively. Although Hep3B and HepG2 were treated with different concentrations of FC, the LC3 staining images between the treatment of FC alone and FC with Baf indicates that Baf further elevated the number, size, and/or intensity of LC3 puncta in both cell lines, demonstrating that accumulation of LC3 occurred in the presence of Baf. It is noteworthy that the increased yellow puncta to a greater extent in FC-treated HepG2 cells in the merged image of LC3 (green) with FTH1 (red; Figure 4D) indicates that FC induced greater ferritinophagy in HepG2 cells compared to that in Hep3B cells.

**Figure 5:**
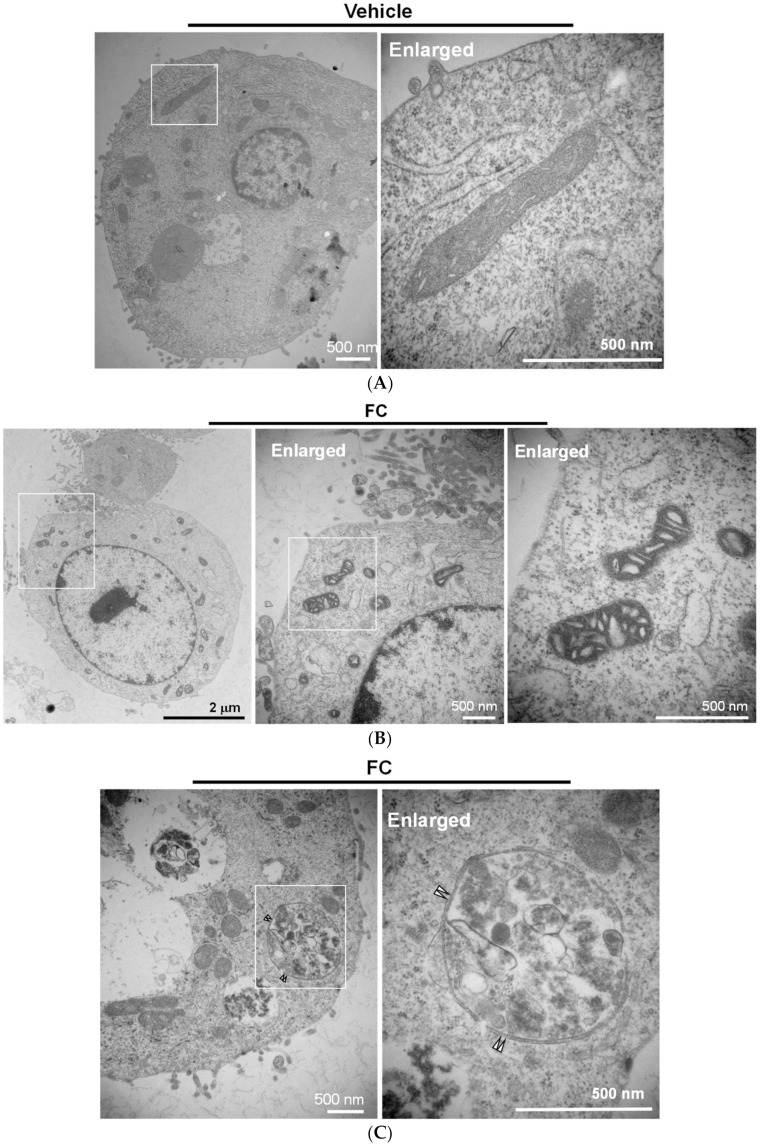
Ultrastructure of FC-treated HepG2 cells. (**A**) Mitochondrial morphology of untreated cells. (**B**) FC increased mitochondrial membrane density. (**C**) FC-induced autophagic vesicle with a double-membrane structure. HepG2 cells were treated with FC (5 µM) for 24 h. The images were taken by electron microscopy. The paired triangle indicates autophagosomes.

**Figure 6:**
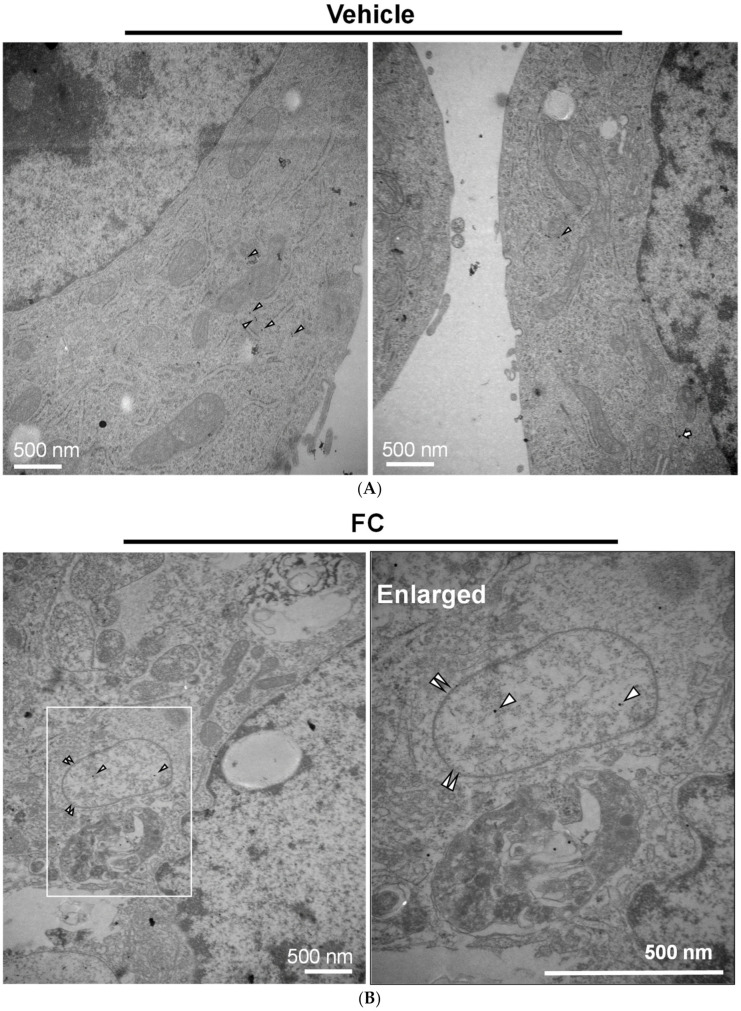

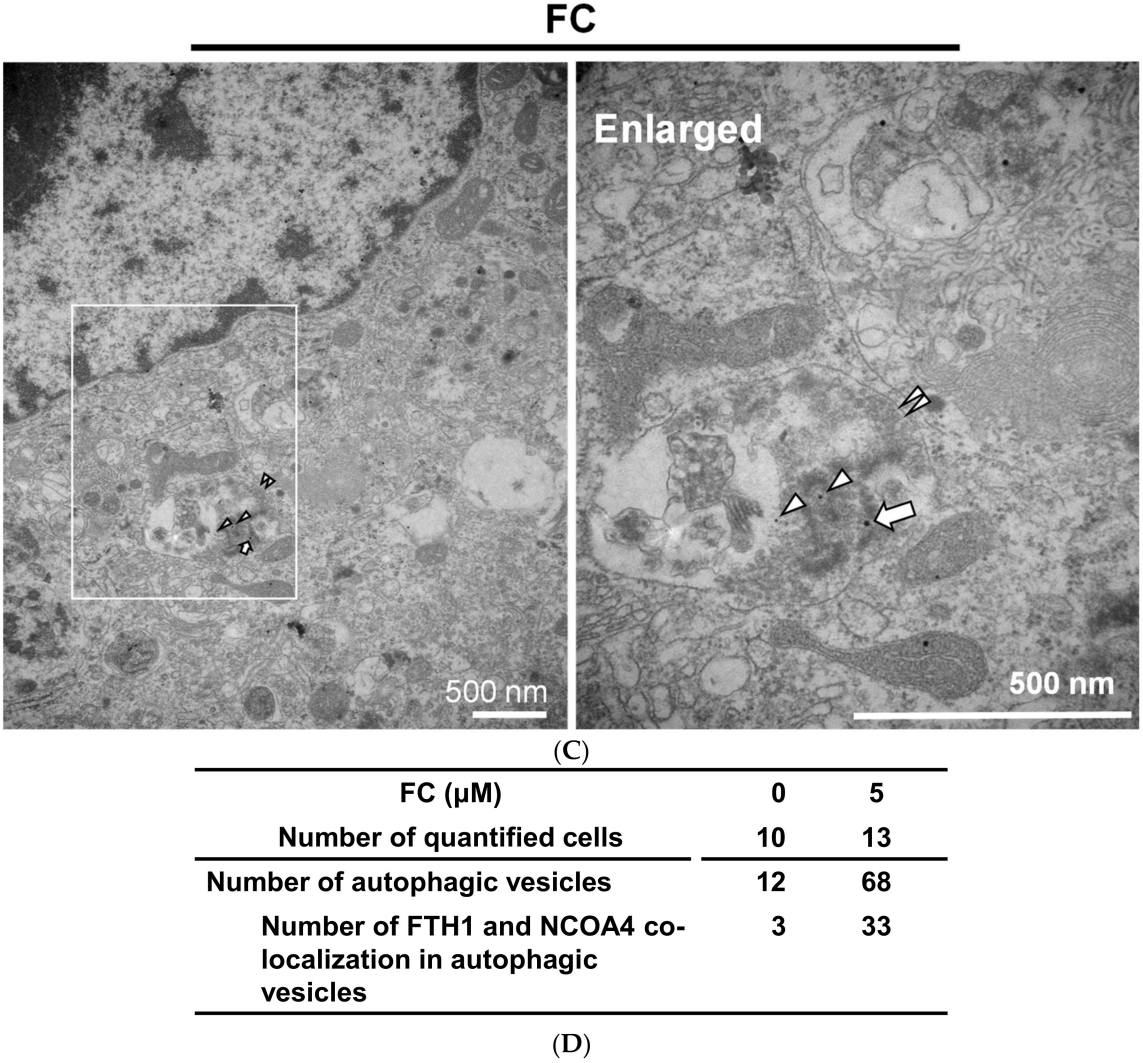
Immunogold-labeled NCOA4 and FTH1 in HepG2 cells. (**A**) The location of NCOA4 and FTH1 in the untreated cells. (**B**) NCOA4 was engulfed in a double membrane vesicle of FC-treated cells. (**C**) FC-induced co-localization of FTH1 and NCOA4 in a double membrane vesicle. (**D**) Quantitation of autophagosomes/autolysosomes and gold particles. More than 10 cells were quantified in each experimental condition. The triangle, arrow, and paired triangle indicate NCOA4 (12 nm), FTH1 (20 nm), and autolysosomes/autophagosomes, respectively.

Transmission electron microscopy analysis was performed to further confirm the FC-induced ferroptosis and ferritinophagy in HepG2 cells. As shown in Figure 5A,B, cytological changes of ferroptotic cells, including the distinctive mitochondrial morphology with increased membrane density [21,34], were displayed in FC-treated HepG2 cells compared to the non-treated control cells. Moreover, the formation of double-membrane vesicles containing subcellular materials, representing the formation of autophagosomes, was seen in the FC-treated cells (Figure 5C and Figure 6D) compared to the non-treated control cells (Figure 5A). These data demonstrate that FC indeed induced ferroptosis and autophagy in HepG2 cells. To confirm the FC-induced ferritinophagy, the samples were labeled with immunogold. As shown in Figure 6A, FTH1 and NCOA4 were located in the cytoplasm of the non-treated cells. Upon FC treatment, the engulfment of NCOA4 (Figure 6B,C) and FTH1 (Figure 6C) in the double-membrane vesicle was observed. The quantitation results are shown in Figure 6D. These data further demonstrate that FC indeed induced both ferritinophagy and ferroptosis in HepG2 cells.

## 4. Discussion

Using ferroptosis-specific inhibitor Ferro-1 to screen various kinds of phytochemicals revealed that FC significantly inhibited the viability of two HCC cell lines via ferroptosis. FC is a diosgenin saponin 3-*0*-α-L-rhamnopyranosyl-(1→4)- α-L-rhamnopy-ranosy-l-(1→4)-[α-L-rhamnopyranosyl]-(1→2)-ß-D-glucopyranoside, isolated using methanol from the dried leaves of *Paris formosana* Hayta (Liliaceae), which is a perennial herb grown in the humid and dark forests of Taiwan at about the level of 1000 m and has been used as a traditional Chinese herbal remedy for the treatment of snake venom poisoning and cyst [35]. Saponins are also readily available food ingredients found in various edible plants, such as soybeans and chickpeas [36]. Recent studies suggest that saponins and FC possess immunological, anti-inflammatory, anti-bacterial, and anti-cancer properties [35,37,38,39,40,41]. FC is also a component of “Yunnan Bai Yao” [42], a traditional Chinese hemostatic medicine. Our previous study showed the apoptotic effect of FC in colorectal cancer HT-29 cells [9]. Our unpublished results also show that FC triggered apoptosis in both Hep3B and HepG2 cells. Since ferroptosis is a non-apoptotic form of cell death, ferroptosis inducers may have unique clinical applications against certain cancer cells that are highly resistant to apoptosis.

FC-induced ferroptosis via ferritinophagy was observed in both HCC HepG2 and Hep3B cell lines. In Figure 1C,D, it is noteworthy that ferroptosis was induced to a greater extent in HepG2 cells, which may be due at least in part to the different state of p53 in these two cell lines, in addition to the opposite expression pattern of NCOA4 and FTH1 (Figure 3C). Several genes and proteins have recently been demonstrated to be involved in ferroptosis, such as p53 and SAT1, which serve as positive regulators, and SLC7A11 and nuclear factor erythroid 2-related factor 2 (Nrf2), which function as negative regulators [43]. Hep3B is p53 null, while HepG2 is p53 wild-type. *TP53* is the most commonly mutated tumor suppressor gene in human cancers. About 12 to 48% of HCC patients have altered expression or mutations of *TP53,* which are associated with a poor prognosis [44]. Recent studies indicate that nuclear p53 is required for ferroptosis in a transcription-dependent manner, whereby nuclear p53 suppresses the expression of SLC7A11, a key component of the cystine/glutamate antiporter, which provides adequate concentrations of cystine (the oxidized form of cysteine) for the synthesis of the antioxidant glutathione, and therefore increases lipid peroxidation [45]. In this regard, cells with p53 may be more sensitive to ferroptosis, which is consistent with our observation. Our Western blotting results (data not shown) also indicate that the expression level of SLC7A11 in the HepG2 cells was indeed lower than that in Hep3B cells, suggesting the role of p53 in the sensitivity of FC-induced ferroptosis. In addition, the Hep3B cell line contains an integrated hepatitis B virus genome and expresses the hepatitis B surface antigen, whereas the HepG2 does not (American Type Culture Collection). Our unpublished data show that FC seems to induce ferroptosis more effectively in HepG2 cells compared to that in HepG2.2.15 cells, which has been reported to readily produce high levels of hepatitis B virus DNA [46], suggesting that the induction of a greater extent of ferroptosis in HepG2 cells by FC may also be due to the different state of hepatitis B virus DNA.

Nrf2 is usually degraded via the proteasomes by binding with Kelch-like ECH-associated protein 1 (Keap1); being a transcription factor, it regulates the expression of several antioxidant genes [47]. Recently, p62 has been reported to compete Keap1 biding with Nrf2 in sorafenib resistant liver cancer cells, leading to an increase in the transcription of antioxidant genes to inhibit ferroptosis [48]. Compared to Hep3B cells, although HepG2 expressed a higher level of p62, it was suppressed by the treatment of FC (data not shown), suggesting that FC may sensitize HepG2 for ferroptosis by decreasing p62. This phenomenon was not observed in Hep3B cells.

By using gene expression databases of CTRP and PRISM, we linked the differences between Hep3B and HepG2 cell lines to the induction of ferroptosis and have successfully predicted a possible mechanism on the expression level of FTH1. Further experiments not only confirmed the results obtained from the databases but also demonstrated that NOCA4, the specific cargo receptor for ferritinophagy, plays a critical role in FC-triggered ferroptosis of Hep3B and HepG2 cell lines. As the correlation was calculated based on all HCC cell lines stored in the databases, the findings in the present study await further investigation on other HCC cell lines.

Acyl-CoA synthetase long-chain family member 4(ACSL4) catalyzes the formation of active acyl-CoA ester from fatty acid for lipid synthesis and ß-oxidation. Recently, it has been revealed that ACSL4, by forming lipid peroxidation substrate arachidonic acid or adrenic acid-containing phosphotidylethanolamine, is essential for the induction of ferroptosis [49]. In addition to regulating the synthesis and degradation of lipids, ACSL4 has been found overexpressed in several origins of cancer [50,51,52]. It is noteworthy that ACSL4 favors tumor progression in HCC via stabilization of the oncoprotein c-myc through the ubiquitin-proteasome system [53], and the mRNA expression of *ACSL4* in HepG2 cells was substantially higher than that in Hep3B cells [53] which is in accordance with the gene expression pattern obtained from the Cancer Cell Line Encyclopedia database (data not shown). Therefore, the higher sensitivity of HepG2 cells to FC-induced ferroptosis may be, at least in part, attributed to the higher expression of ACSL4.

Ferritin is composed of ferritin heavy chain and ferritin light chain. The heavy chain catalyzes the first step in iron storage, the oxidation of Fe^2+^; whereas the light chain promotes the nucleation of ferrihydrite, enabling storage of Fe^3+^. Previous studies showed the expression of ferritin is down-regulated in ferroptosis-sensitive cells compared to ferroptosis-resistant cells [20]. These observations suggest that ferritin negatively regulates ferroptosis, which are consistent with our database searching results (Figure 3A,B) and our experimental data. Although the degradation of FTH1 was prevented in the presence of Baf to block the autophagic degradation activity, increased expression of FTH1 was displayed in both cell lines treated with FC (Figure 3C). This phenomenon may be due to a cellular labile iron accumulation during ferroptosis to induce transcriptional up-regulation of endogenous ferritin heavy chain [4]. It has been known that chemotherapeutic agents elevate ROS formation. However, drug-resistant cells develop several ways to avoid the detrimental effects of ROS, especially increasing glutathione levels. Thus, any disruption in these processes could induce ferroptosis. As a ROS-dependent cell death mechanism, the FC-increased intracellular level of ROS and -induced ferroptosis may provide a new alternative for treating HCC.

In conclusion, our results discovered that saponin FC is a novel natural ferroptosis inducer, which triggered a stronger ferroptosis in human HCC HepG2 cells containing a higher level of NCOA4 and a lower level of FTH1 compared to Hep3B cells. A lower expression of FTH1 in HepG2 cells indicates higher basal autophagy in the cells and leads the HepG2 cells to be more responsive to FC-induced autophagy and ferritinophagy.

## Abbreviations

ACSL4, acyl-CoA synthetase long-chain family member 4; AVOs, acidic vesicular organelles; Baf, bafilomycin A1; CTRP, Cancer Therapeutics Response Portal; Ferro-1, ferrostatin-1; FC, formosanin C; FPN, ferroportin; TFRC, transferrin receptor; FTH1, ferritin heavy chain 1; GFC, garcinielliptone FC; HCC, hepatocellular carcinoma; Keap1, Kelch-like ECH-associated protein 1; MTT, 3-[4,5-Dimethylthiazol-2-yl]-2,5-diphenyltetrazolium bromide; NCOA4, nuclear coactivator 4; Nrf2, nuclear factor erythroid 2-related factor 2; PRISM; Profiling Relative Inhibition Simultaneously in Mixtures; ROS; reactive oxygen species; SEMs; standard errors of the means; SRB; sulforhodamine B.
